# Ramping activity in the striatum

**DOI:** 10.3389/fncom.2022.902741

**Published:** 2022-08-01

**Authors:** Adam Ponzi, Jeff Wickens

**Affiliations:** ^1^Institute of Biophysics, Italian National Research Council, Palermo, Italy; ^2^Neurobiology Research Unit, Okinawa Institute of Science and Technology (OIST), Okinawa, Japan

**Keywords:** interval discrimination, computational model, striatum, basal ganglia, neural ramping, network dynamics, temporal estimation

## Abstract

Control of the timing of behavior is thought to require the basal ganglia (BG) and BG pathologies impair performance in timing tasks. Temporal interval discrimination depends on the ramping activity of medium spiny neurons (MSN) in the main BG input structure, the striatum, but the underlying mechanisms driving this activity are unclear. Here, we combine an MSN dynamical network model with an action selection system applied to an interval discrimination task. We find that when network parameters are appropriate for the striatum so that slowly fluctuating marginally stable dynamics are intrinsically generated, up and down ramping populations naturally emerge which enable significantly above chance task performance. We show that emergent population activity is in very good agreement with empirical studies and discuss how MSN network dysfunction in disease may alter temporal perception.

## Introduction

The basal ganglia (BG) are subcortical nuclei that receive input from almost the entire cortex and thalamus. Multiple studies, including from diseases, lesions, and pharmacological or genetic manipulations that affect the BG as well as from fMRI and neurophysiology have demonstrated the involvement of the BG in both sensory and motor timing tasks when behavioral timescales are in the range of hundreds of milliseconds to several seconds (Paton and Buonomano, 2018). This is to be expected because the BG is also well known to be deeply involved in reinforcement learning (Ito and Doya, 2011), which requires prediction of the time of future events, as well as in the planning and execution of behavior which depends on accurate control of action sequence timing.

There are multiple human diseases and pathologies that affect the BG which display aberrant time estimation. Parkinson's disease (Pastor et al., 1992; Malapani et al., 1998), Huntington's disease (Freeman et al., 1996), Tourette's syndrome (Vicario et al., 2010), substance abuse (Wittmann et al., 2007), and ADHD (Noreika et al., 2013) are all diseases of the BG which have been associated with the disrupted estimation of the passage of time or deficits in rhythmic behavior. In healthy humans too, fMRI (Schubotz et al., 2000; Ferrandez et al., 2003; Nenadic et al., 2003), EEG (Pfeuty et al., 2003), and PET (Jahanshahi et al., 2006) studies have all demonstrated sensory and motor timing to be linked to the BG.

The striatum occupies a privileged place in the BG. It is its largest part and its main input structure. It has been particularly implicated in interval timing tasks in multiple species (Harrington et al., 1998; Malapani et al., 1998; Hinton and Meck, 2004; Matell and Meck, 2004; Wencil et al., 2010; Coull et al., 2011; Adler et al., 2012; Merchant and de Lafuente, 2014; Emmons et al., 2016, 2017, 2020; Dallerac et al., 2017) trained to discriminate long and short intervals show significant fMRI activation in the caudate in particular (Rao et al., 2001; Pouthas et al., 2005). Several studies have employed striatal inactivation by lesions or pharmacological and genetic manipulations and have shown that doing so disrupted the animal's estimate of elapsed time and impaired performance (Drew et al., 2003, 2007; Meck, 2006; Gouvea et al., 2015). These studies suggest that normal functioning of the striatum is required for time dependent behavior.

How the striatal neurons represent elapsed time is an open question, however. Some striatal medium spiny neurons (MSNs) employ “time ramping” activity. This is a monotonic change in firing rate, which can be either increasing or decreasing, over the temporal interval which needs to be estimated. Such a ramping mechanism could be exploited by the brain to encode elapsed time (Matell et al., 2003; Parker et al., 2014; Donnelly et al., 2015; Gouvea et al., 2015; Mello et al., 2015; Emmons et al., 2017, 2020; Kim et al., 2018). As much as a third of striatal neurons exhibit such ramping activity (Emmons et al., 2017). However, even though recent studies show striatal activity topographically reflects cortical activity (Peters et al., 2021), striatal ramping activity does not seem to depend on cortical ramping activity (Emmons et al., 2019, 2020). Indeed while inactivation of the medial frontal cortex does attenuate striatal ramping (Emmons et al., 2017, 2019), fixed magnitude non-ramping corticostriatal stimulation is sufficient to recover the decreases (Emmons et al., 2019). Moreover, many ramping neurons do not show significant lever-pressing activity (Emmons et al., 2017) suggesting their role is predominantly time estimation, rather than control of the action.

More generally ramping is just one example of a neural mechanism for time estimation that employs continuously evolving population dynamics as a general mechanism for time encoding across the brain (Buonomano and Merzenich, 1995; Mauk and Buonomano, 2004; MacDonald et al., 2011; Gershman et al., 2013; Buonomano, 2014; Paton and Buonomano, 2018). According to this view, time may be encoded by any reproducible pattern of activity across a population of neurons, as long as the pattern is continuously changing and non-repeating. Indeed studies of striatal MSN dynamics in interval tasks using principal component analysis have shown that ramping activity is only the first component, the next two or three higher components are oscillatory and also explain significant activity variance (Emmons et al., 2017, 2020). In agreement with this viewpoint striatal MSNs exhibit varied and diverse temporal response profiles which activate at particular delays after task events and can, therefore, be used to encode elapsed time. Such activity has been found in monkey sequential saccade tasks (Jin et al., 2009), in rodents during locomotion (Rueda-Orozco and Robbe, 2015), in rats trained to press a lever for a reward delivered on a fixed interval reinforcement schedule (Matell et al., 2003; Dhawale et al., 2015; Gouvea et al., 2015; Mello et al., 2015), and mice trained to lick for reward delivered after a fixed delay (Bakhurin et al., 2017) for example. Strong support for a direct causal role for MSNs in time control was found in a recent study of an interval categorization task (Gouvea et al., 2015). The authors were able to decode trial-by-trial variations in duration judgments from the activity of simultaneously recorded ensembles of striatal MSNs. Interestingly animals were more likely to categorize an interval as being long (short) on trials where striatal activity progressed faster (slower) than normal. MSN response profiles can even show temporal rescaling when task time intervals are changed (Mello et al., 2015; Murray and Escola, 2017).

In previous computational modeling of MSN network dynamics (Ponzi and Wickens, 2008, 2010; Ponzi et al., 2020) we showed that, when network parameters are appropriate for the striatum, MSN cells spontaneously form cell assemblies that inhibit each other and are activated sequentially on behaviorally relevant timescales, the importance of which has been investigated by several other studies (Humphries et al., 2009; Angulo-Garcia et al., 2016; Spreizer et al., 2017). We were also able to show that when the network was driven by temporally varying cortical input (Ponzi and Wickens, 2012, 2013; Ponzi, 2017), as would be expected to occur in behavioral and cognitive tasks, the elapsed time between task events could be decoded from MSN population activity and that individual MSN cells activated consistently and reproducibly at particular temporal delays after task events, in good agreement with empirical studies (Jin et al., 2009). We found population activity in such tasks as described by three or four dominant principal components with a high cross-trial signal-to-noise ratio (Ponzi and Wickens, 2013). Here, we extend this model to include a simple mechanism for animal behavioral choice. We apply the model to the interval discrimination task described in Gouvea et al. (2015). We recover extremely good agreement with their findings including the emergence of increasing and decreasing ramping populations (Emmons et al., 2017, 2020) which cross-over close to the discrimination choice boundary. We discuss how dysfunction in MSN network activity can be expected to alter time estimation in pathologies such as HD (Ponzi et al., 2020).

## Results

Here, we investigate a simple model of an interval discrimination task similar to the one studied in Gouvea et al. (2015). In this task, the animal must choose one of two responses dependent on whether an interval is relatively long or short. In the task described in Gouvea et al. (2015), in each trial, the rat initiates a trial with a nose-poke which generated a 150 ms auditory tone. This is followed by a variable length silent interval, after which another 150 ms auditory tone sounds and the animal immediately reports a long or short judgment. Silent intervals had lengths randomly chosen from 600, 1,050, 1,260, 1,380, 1,620, 1,740, 1,950, and 2,400 ms and short (long) ones are those shorter (longer) than 1,500 ms. In the task studied in Gouvea et al. (2015) if the response is correct the rat gets a water reward, if not a white noise sound signals a time-out.

We use a previously published model of the spiking inhibitory MSN network (Ponzi and Wickens, 2010, 2012, 2013) to represent the striatal activity. MSNs are represented by a conductance based spiking point neuron cell model with MSN-like characteristics. In particular, the cell model shows a Type I transition to firing characterized by a continuous increase in firing rate from zero as input current crosses the threshold. Model cells can, therefore, fire at the very low rates (Izhikevich, 2005) typical of MSNs and do not show sub-threshold oscillations (Nisenbaum and Wilson, 1995; Wilson and Kawaguchi, 1996).

Cells are connected together by Rall type synapses (Rall, 1967) including a fairly slowly decaying inhibitory neurotransmitter, modeling the GABAergic action of MSNs on each other. The MSN network does not include any form of synaptic plasticity, either short term or (anti-)Hebbian. In this simplest model, we also do not include the striatal interneurons. In particular, the action of the most important group of interneurons, the fast spiking interneurons, is thought to be entirely feed-forward. Therefore, their action is here considered simply to be included in the strength of the cortical excitation.

As in Ponzi and Wickens (2013) the task environment is modeled very simply by changing the excitatory driving activations to the MSN network model. This excitatory driving originates from the cortex and thalamus. Particular sensory stimuli are described by a particular distribution of excitatory activations across all the MSN cells in the network, which are fixed for the duration of the stimulus. Each sensory stimulus is represented by a different random set of activations. We only use two sensory stimuli to model this task. One sensory stimulus represents the auditory cue and is applied for 150 ms at the start and the end of a trial. The other sensory stimulus is applied during the silent interval between the auditory cues and also between trials. One of the eight possible intervals *T* between 600 and 2,400 ms, as described above, is randomly chosen for each trial, as in Gouvea et al. (2015). The inter-trial interval has a random length with a mean of 800 ms. A single network simulation includes many, M, such trials which follow each other in sequence. The number of trials, M, in a single network simulation, varies but is approximately 130, (refer to Methods).

We do not explicitly include reward in this simple model and the selection of whether a trial was long or short by the animal is directly determined by the network activity at the end of the silent interval in the same way as described in Gouvea et al. (2015). First, the fifty cells with the highest average firing rate throughout the whole simulation are selected. The firing rate of each of these 50 cells in the final 500 ms of each trial interval is determined for each of the M trials except one. This gives M-1 points in the 50 dimensional space of the firing rates. Some of these M-1 points are associated with short trials *T* <1, 500, and some with long trials *T*>1, 500, defining two clusters. Next, we use the Fisher Linear Discriminant (FLD) analysis to find the direction which best discriminates these long and short trial clusters. Finally, the classification for the remaining trial which was left out of the calculation is determined by projecting it onto this maximally discriminating direction. This gives the animals choice for that trial. This process is repeated M times, each time leaving out a different trial to calculate the animals' choice for each trial. Finally, the correct response probabilities (CRP) are determined by comparing the animals' choice on each trial with whether the trial interval was actually long or short (refer to Methods).

In previous study (Ponzi and Wickens, 2008, 2010, 2012, 2013; Ponzi, 2017), we have shown that depending on network parameters such as the connection probability, connection strength, and excitatory input strength the network shows different dynamical regimes. At high connection probability, a stable winner-take-all like state is found. Although spiking is random and close to Poissonian, cells fire at fixed rates. Some cells fire at high rates (the winners), others low, and some are quiescent (the losers). On the other hand at low connection probability cell firing rates fluctuate chaotically and this shows up as very bursty spiking activity. In the interface regime, when network dynamics are marginally stable the network dynamics fluctuate slowly and we find MSN cells spontaneously form cell assemblies that inhibit each other and can be activated sequentially on behaviorally relevant timescales. In previous study, Ponzi and Wickens (2012, 2013) and Ponzi (2017), we have found that the maximal Lyapunov exponent, denoted λ, of the network rate dynamics, is a very useful measure of network dynamical behavior and, in particular, how the network responds to variations in cortical driving activity. To isolate the dynamics of the slow rate variations, rather than the details of the spiking, maximal Lyapunov exponents are not calculated from the spiking network itself but the *equivalent rate network*. Pairs of equivalent network simulations, spiking and rate, have identical network structure and task driving inputs except the spiking cell model is replaced by a rate cell model with a similar rheobase and firing rate to input current response curve (Ponzi and Wickens, 2012, 2013) as the spiking cell.

**Figure 1:**
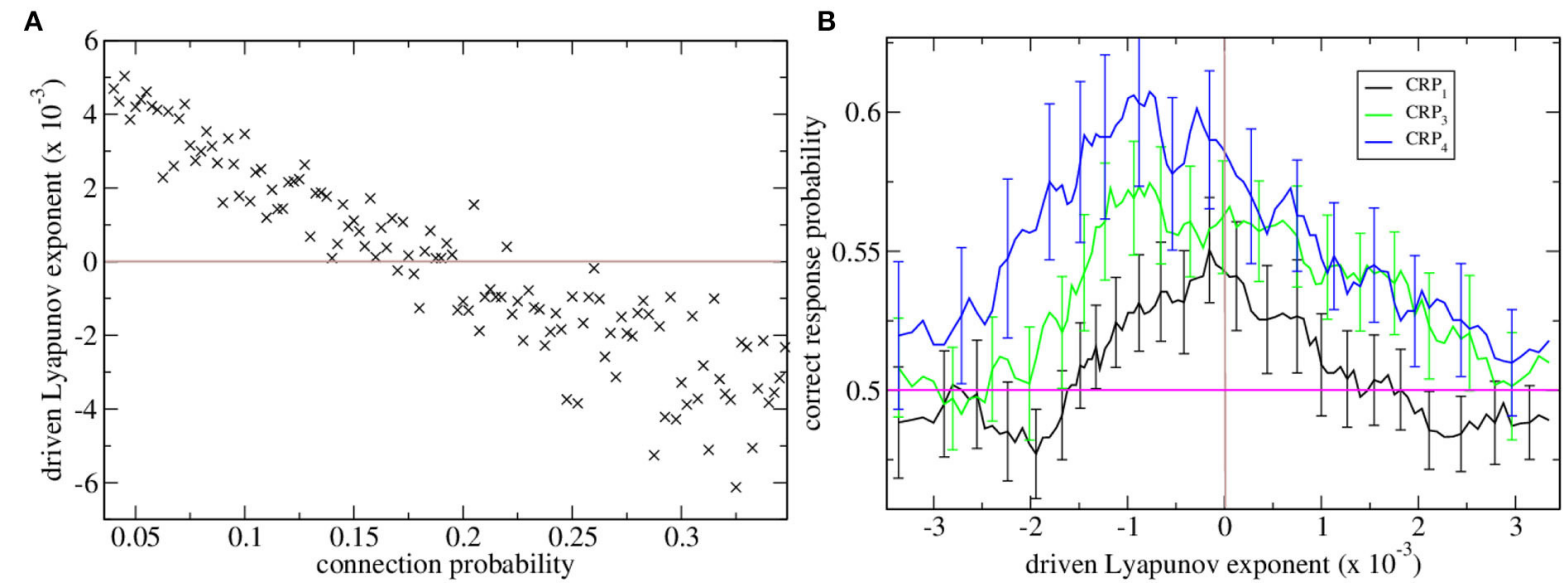
**(A)** Driven maximal Lyapunov exponent λ vs. network connection probability for many network simulations. Each point shows λ for a single network simulation with a given connection probability, ρ. Connection probability ρ increases from 0.04 in increments of 0.0025. The brown horizontal line shows λ = 0 which is the transition from chaotic to stable dynamical behavior. **(B)** Correct response probability (refer to key) vs. driven Lyapunov exponent λ for many network simulations. CRPs are calculated for different length *T* intervals separately, the 600 and 2,400 ms intervals are combined and denoted CRP_4_, (blue) the 1,050 and 1,950 ms intervals are combined and denoted CRP_3_, (green) and the 1,380 and 1,620 ms intervals are combined and denoted CRP_1_ (black). Here, the Lyapunov exponents λ and corresponding CRP values for all the simulations shown in (a) are obtained and re-ordered according to their λ values. 15 point moving averages over adjacent λ values, and the corresponding CRP values are calculated. The solid lines show these mean λ values and the corresponding mean CRP values. The error bars show the SEM in the CRP values at each point. Error bars on the λ values at each point are not shown. The pink horizontal line shows the performance probability of chance.

Maximal Lyapunov exponents are calculated in the standard way from the dynamical evolution of two identical rate network simulations with slightly different initial conditions. Here, we are concerned with the *driven* maximal Lyapunov exponent. This is the maximal Lyapunov exponent of a non-autonomous dynamical system with inputs that vary in time, as in the task described here. In this case, to calculate λ we use two identical rate network simulations driven by identical sequences of cortical driving but with slightly different initial conditions. λ measures how fast trajectories from these two systems diverge or converge from each other. When λ is positive the driven network dynamics are chaotic, when it is negative the dynamics are stable and when it is close to zero the dynamics are marginally stable. The variation in λ with connection probability is shown in Figure 1A. When λ is positive the network rate dynamics is chaotic and strongly fluctuates but when it is negative the network rate dynamics finds a stable equilibrium fixed point. The transition between stable and chaotic network dynamics at certain connectivity around 0.17 is clearly seen.

Here, we find network stability is an important determinant of interval classification performance. CRPs are shown in Figure 1B vs. network stability measured by the Lyapunov exponent. The variation of the Lyapunov exponent λ occurs because we have generated many network simulations by varying the network connection probability. As described above the Lyapunov exponent λ is calculated from the rate network while the CRPs are calculated from the matching spiking network.

Correct response probabilities are divided into CRPs for least discriminable, i.e., those closest to the 1,500 ms short/long boundary, intermediate and most discriminable trial intervals. CRPs clearly increase with interval discriminability throughout the range of network stabilities, λ, and peak close to the ‘edge of chaos’, λ≈0, where dynamics is just stable, Figure 1B. This ‘edge of chaos’ regime is also the regime of realistic connection probability for the striatum (Ponzi and Wickens, 2010). It also seems that the peak CRP location approaches the edge of chaos as intervals become less discriminable.

**Figure 2:**
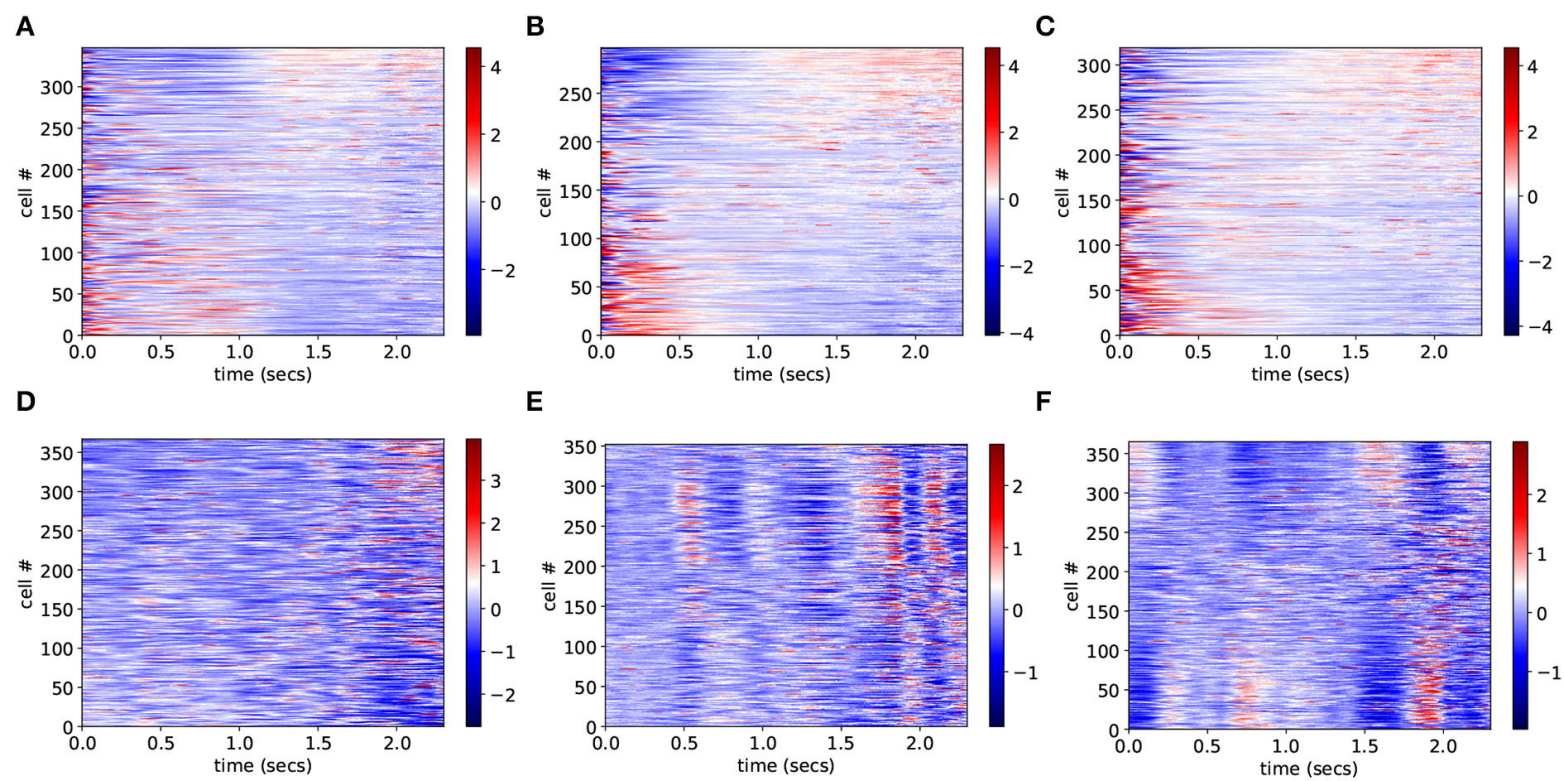
**(A–F)** PSTH of firing rates z-scores for cells in six different network simulations. Statistics are shown in Table 1. Firing rates are binned at 10 ms and then further smoothed with a 10-point moving average. Time indexes the onset of the moving average, so the minimum is zero and the maximum is 2.3 s. Cells are ordered by preference indices from short preferring to long preferring. All active cells with absolute preference indices over 0.02 are included. Results are averages over all intervals, *T*, so bins at later times include fewer observations.

To investigate why CRPs peak in this marginally stable regime, we calculate preference indices for each cell in the spiking network simulation as in Gouvea et al. (2015). To calculate preference indices we compare the distribution of firing rates during the final 500 ms on trials where the animal makes a short choice with the distribution of firing rates during the final 500 ms on long choice trials for each cell separately using Receiver Operant Characteristic (ROC) analysis. Preference indices are ROC z-scores which are obtained from the ROC values for each cell by comparing the calculated values with those generated from surrogate firing rate distributions where animal choice sequences are scrambled. Figure 2 shows cell firing rate z-score peri-stimulus time histograms (PSTH) for several exemplar model simulations. Only cells with a short/long preference are included and cells have been ordered by their preference indices, from long to short preferring. Here, the cell firing rates are averaged across trials including all intervals *T* to obtain PSTH with interval onset at time zero. Since cells have different baseline firing rates the cell PSTH is standardized by subtracting their means across the 2,400 ms and dividing by their SDs.

In order to illustrate network dynamics, Figures 2A–C shows some exemplar model simulations with relatively high CRP_4_ values. The corresponding network stabilities and connection probabilities are shown in Table 1. All three simulations have very small slightly negative λ demonstrating that the rate dynamics of the corresponding rate network simulation are marginally stable. These networks are quite sparsely connected and in the striatally relevant regime. In general, if a network has a relatively high CRP_4_ value it will also have relatively high values for the other CRP measures, but this is not always the case. For example network (a) has a CRP_4_ value that exceeds network (c) but its CRP_3_ value does not. CRP values, in general, decrease from CRP_4_ to CRP_1_ as the interval pairs become less discriminable, as expected. Evidently, in these simulations, Figures 2A–C, firing rates for long preferring cells gradually increase throughout the 2,400 ms interval, while the converse is true for short preferring cells. Three other examples of various connection probability and network stability are shown in Figures 2D–F. These have much lower CRP values, Table 1. The slow ramping activity shown in Figures 2A–C is absent in these examples. Instead, activity seems to be much more rapidly fluctuating. Only the examples with marginal stable dynamics, Figures 2A–C, seem similar to the results shown in Gouvea et al. (2015) (Figure 2D). These examples are also very similar to the ones shown in Emmons et al. (2017) (**Figure 4D**) and Emmons et al. (2020) (**Figures 4D**, **5F**).

**Table 1:** Results for Figure 2.

**Panel**	**Connectivity**	**λ**	**CRP_1_**	**CRP_2_**	**CRP_3_**	**CRP_4_**
(a)	0.1775	–0.000331341	0.565217	0.641026	0.676471	0.8125
(b)	0.2425	–0.00140198	0.529412	0.357143	0.692308	0.888889
(c)	0.21	–0.000961602	0.578947	0.571429	0.785714	0.75
(d)	0.0625	0.00228291	0.526316	0.607143	0.466667	0.5
(e)	0.345	–0.00316362	0.391304	0.538462	0.411765	0.53125
(f)	0.2275	–0.00214502	0.5	0.571429	0.5	0.53125

**Figure 3:**
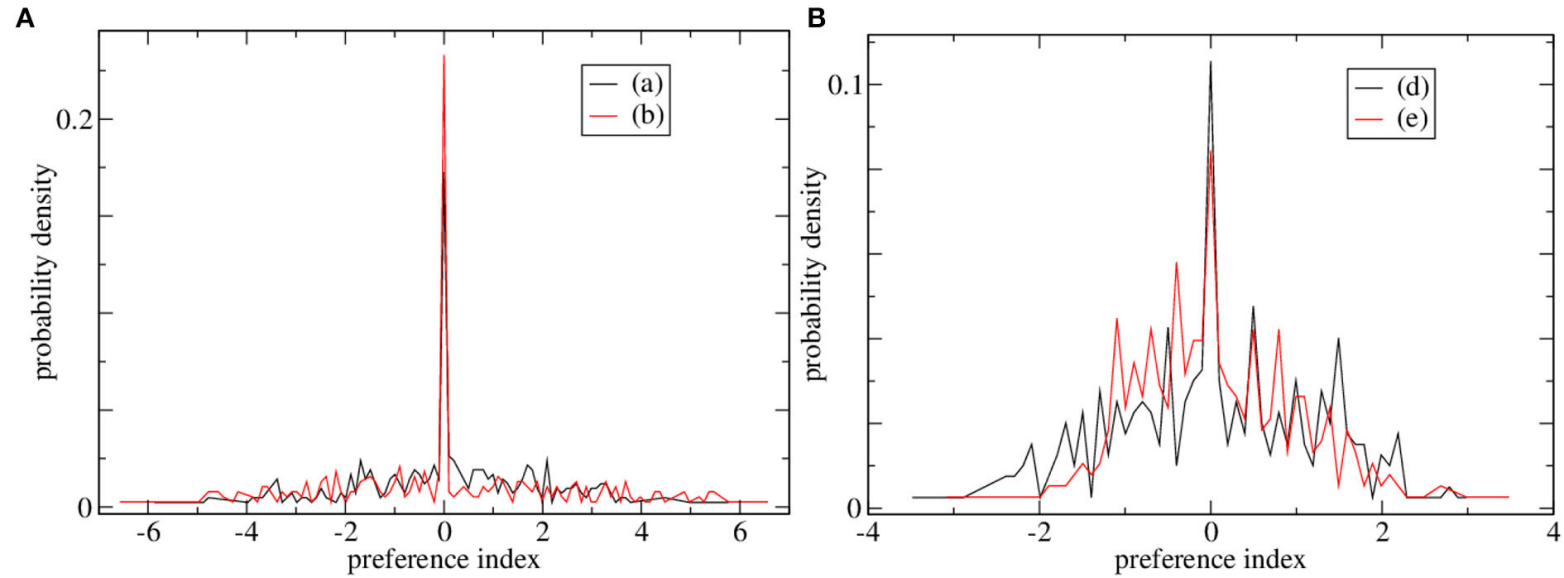
Distributions of preference indices for four of the simulations shown in Figure 2. **(A)** Two simulations with high CRP_4_, (black) Figure 2A and (red) Figure 2B. **(B)** Two simulations with low CRP_4_, (black) Figure 2D and (red) Figure 2E.

Preference index distributions for two of the high CRP marginally stable simulations and two of the low CRP simulations are shown in Figures 3A,B, respectively. The distribution of preference indices is much broader for the high CRP simulations, Figure 3A than the low CRP ones, Figure 3B. Such broad ‘long-tailed’ distributions arise in critical and marginally stable systems in general. The presence of cells with high absolute preference index values indicates that ramping activity with a ramping direction preference which is consistent across trials is present. In these high CRP simulations, there are some cells with strong absolute preferences and also a peak at zero indicating many cells with no preference. This division into discriminating and non-discriminating MSNs may also be in some agreement with empirical studies. On the other hand, no such division is evident in the preference distributions for cells in the low CRP network simulations, Figure 3B, which appear much more Gaussian. The low CRP simulations, Figure 3B, do not contain cells with large absolute preference indices, i.e., cells with strong positive or negative ramping activity.

**Figure 4:**
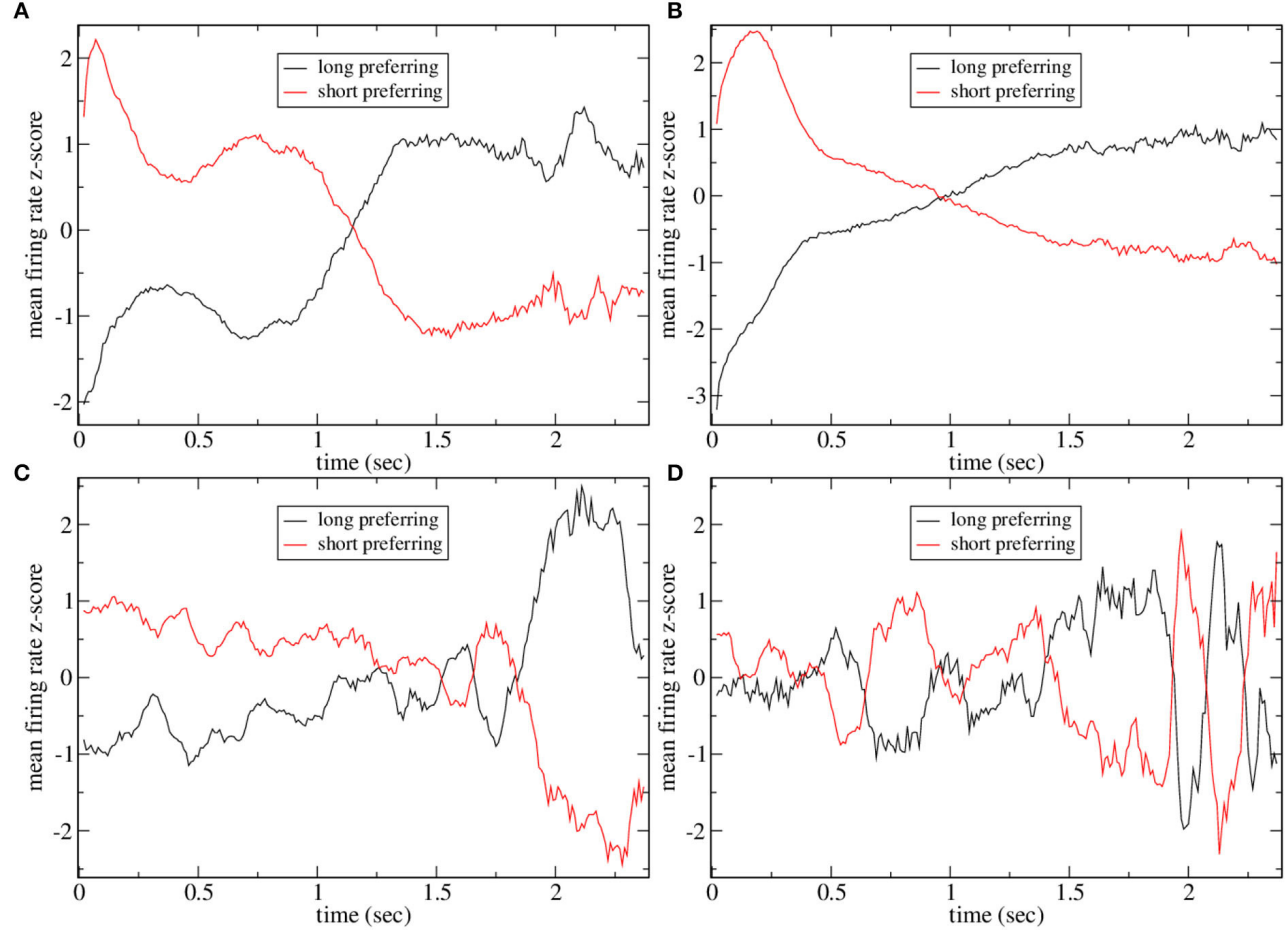
Mean firing rate z-scores for all short and long preferring cells with an absolute preference index exceeding unity for some of the simulations shown in Figure 2. **(A)** Figure 2A, **(B)** Figure 2C, **(C)** Figure 2D, and **(D)** Figure 2E. Averages include all intervals *T* so points at later times are averaged over fewer observations and are expected to be noisier. Five point moving averages.

**Figure 5:**
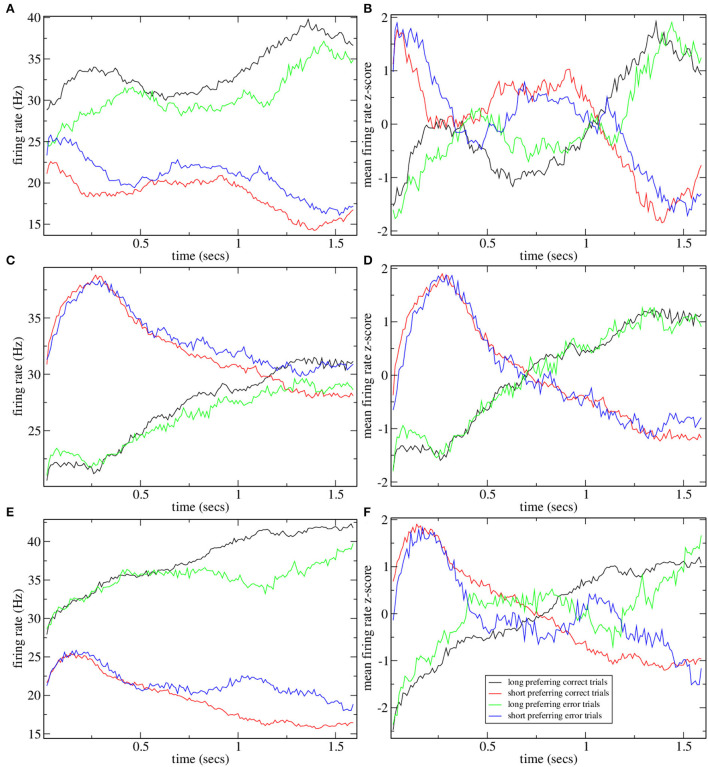
Mean firing rate **(A,C,E)** and z-scored mean firing rate **(B,D,F)** for all short and long preferring cells with an absolute preference index exceeding unity for some simulations shown in Figure 2 on *T* = 1620 ms trials further divided into whether the trial was classified as long (i.e., correct) or short (i.e., error). Five point moving averages. **(A,B)** Figure 2A, **(C,D)** Figure 2B, **(E,F)** Figure 2C.

Figure 4 shows the mean firing rate z-score PSTH for all significantly long and short preferring cells (with the absolute value of preference index exceeding unity) for several simulations shown in Figure 2. The two examples with high CRP, Figures 4A,B, clearly demonstrate the ramping activity with cross-over close to the dividing point between long and short trials, *T* = 15, 00, resembling the mean firing rate z-scores in Gouvea et al. (2015) (Figure 2F). The two examples, Figures 4C,D, with low CRP do not exhibit ramping activity. Finally, in Figure 5 we show time series of mean firing rates and mean firing rate z-scores for significantly long and short preferring cells but only on intermediate length trials *T* = 1, 620 and further divided into correct (i.e., long choice) and error (i.e., short choice) trials. Only the three simulations in Figure 2 with high CRP are shown. Ramping activity is evident in both the z-scores Figures 5B,D,F and the firing rates themselves Figures 5A,C,E. In all three cases, the ramping activity of the firing rate Figures 5A,C,E is weakened on error trials compared to correct trials. At the end of the interval long preferring cell populations have higher firing rates on correct compared to error trials while short preferring populations have lower firing rates on correct compared to error trials. This could also be seen as an increase in the average ramping rate on correct compared to error trials. This is reflected in the z-scores as a less clear transition from excess short preferring to excess long preferring at around *T* = 1, 500, in qualititative agreement with Gouvea et al. (2015) (Figures 2G,H). Note that response is determined from the firing rates in the last 500 ms of the *T* = 1, 620 trial. While the example in Figure 5C does indeed show that the firing rate of short preferring cells exceeds that of long preferring cells on error trials, in contrast, to correct trials, akin to the example in Gouvea et al. (2015) (Figures 2G,H), in the examples shown in Figures 5A,E, the difference between short and long preferring activity is simply weakened on error trials compared to correct trials. There are several factors involved in this. First, the choice decision employs FLD analysis to find the most discriminative direction. This direction is not simply determined by the mean activity of the cells in the final 500 ms on short and long trials but also depends on the cross trial covariance (refer to Methods). Second, the FLD analysis uses only the fifty cells with the highest average firing rates. These fifty cells do not necessarily coincide with the significantly short or long preferring cells which are included in the activity shown in Figure 5. Third, cell preference indices are calculated based on trial intervals *T* of all lengths, while the results in Figure 5 include only *T* = 1, 620 trials.

## Discussion

We investigated the behavior of an MSN network model combined with a choice selection system in a temporal discrimination task. We found that only when the network generated marginally stable dynamical evolution correct response probabilities even for the least discriminable intervals were substantially above chance level. Indeed when the Lyapunov exponent is just below zero, mean CRP_1_ values, Figure 1B, appear to be several standard errors above 0.5. Such critical dynamics are generated when the network is quite sparsely connected in agreement with studies of actual connectivity in the striatum. We found that in this regime many cells showed strong preferences for long or short trials associated with the emergent ramping activity. The ramping activity of the ramping population was aberrant on error trials leading to mis-classification of the interval. We closely replicated the task and data analysis employed in Gouvea et al. (2015) and found excellent agreement with their results, particularly the ramping activity of the long and short preferring cells. The ramping activity we found was also in good agreement with Emmons et al. (2017, 2020). Our results did not require any form of plasticity either between the MSN cells or on the cortical-striatal synapses. These results are not a trivial result of any inhibitory network dynamics since networks that are not marginally stable have neither high CRPs, Figure 1, nor ramping cells with large preference indices, Figure 3.

There is no learning or plasticity in this model. Ramping activity is simply an outcome of the slow dynamical evolution of the network. Because the rates are varying slowly, there are some cells with significantly higher firing rates at the end of a stimulus interval period compared to the start and some with significantly lower activity than at the start. In each trial, the cue stimulus partially ‘resets’ the network population activity to roughly the same location in 500 dimension population activity space, so that the dynamical evolution during the interval period is roughly consistent across trials. Such properties are found when the dynamics are marginally stable because such dynamics generate large excursions without falling to a stable fixed point attractor too rapidly, but also do not vary so strongly that they are not consistent across trials (Ponzi and Wickens, 2013; Ponzi, 2017). This is sufficient to be informative for several of the empirical results in Gouvea et al. (2015). However, we believe the dynamical behavior described here will be highly relevant and provide insight when combined with plasticity mechanisms in more complex tasks which do require learning across multiple trials. For example in the task described (Mello et al., 2015), time intervals are fixed within a block and only changed across multiple blocks. As a block progresses animals learn the time interval for the current block and learn to press the lever to obtain a reward at the appropriate time. Interestingly, MSN ramping activity profiles are found to rescale according to the interval for the particular block. Although these results cannot be produced in the current model as is, one can easily imagine that the ramping activity shown here could be combined with secondary mechanisms, e.g., cortical-striatal plasticity developing over multiple trials as the reward is received, which increases excitatory drive and changes the ramping rate.

Indeed, we did not fully implement an action selection reinforcement learning system but applied the Fisher Linear Discriminant methodology as it is used in Gouvea et al. (2015). It is easy to see that the ramping populations generated by this network could be exploited by a full action learning system. For example, one could imagine that downstream of the striatum there are two mutually inhibitory populations (or even single cells), denoted *S* and *L* generating the short and long choice actions. At first, one or an other of these populations is activated randomly. However, once the correct action occurs and reward is generated, reward modulated Hebbian plasticity could link the striatal MSN cells active at the end of the trial when the reward is received with the activated downstream action population. If *L* is active so the long action choice occurs and it is correct then the strongly active long preferring MSN cells would be linked to the downstream long action generating population, *L*. On the other hand, if *S* is active so the short action choice occurs and it is correct then the strongly active short preferring MSN cells would be linked to the downstream short action generating population, *S*. After multiple trials of learning the MSN population ramping activity would influence the downstream activity resulting in a gradual increase in the activity of the *L* population and decrease in activity of the *S* population as a trial interval progressed.

This model may provide insight into the aberrant timing behavior which has been associated with BG dysfunction in various diseases such as HD, PD, and schizophrenia (Buhusi and Meck, 2005; Buhusi and Cordes, 2011; Snowden and Buhusi, 2019). Striatal medium spiny cell loss and striatal atrophy are characteristics of HD (Graveland et al., 1985; Vonsattel and DiFiglia, 1998; McColgan et al., 2017). HD (Paulsen et al., 2004; Beste et al., 2007; Cope et al., 2014; Rao et al., 2014; Righi et al., 2016; Agostino et al., 2017) and sometimes pre-HD (Paulsen et al., 2004; Beste et al., 2007; Zimbelman et al., 2007; Rao et al., 2014) patients show impaired temporal processing. Patients with HD tend to have difficulty with relative timing and interval discrimination, as in the current task, which is typically associated with the striatum (Lemoine et al., 2021) in contrast to absolute timing where a learned interval has to be produced which is often associated with the cerebellum (Grahn and McAuley, 2009; Grube et al., 2010; Teki et al., 2011). In recent work, the parameters of the MSN network model employed here were estimated from spiking data from WT and genetically modified HD mice (Ponzi et al., 2020). The best fit HD model was characterized by stronger MSN network inhibition and weaker cortical driving excitation than the best fit WT model. Such parameter changes, in particular, the change in MSN network inhibitory strength moves the network away from the critical marginally stable regime and is associated with a loss of dynamical complexity (Ponzi et al., 2020). As demonstrated in Figure 1 movement out of the marginally stable regime would also be expected to impair temporal discrimination performance in agreement with empirical studies of HD.

Although PD is well-known to involve aberrant temporal processing (Allman and Meck, 2012), and PD patients show both interval timing and motor rhythm timing deficits (Harrington et al., 1998), timing problems in PD are not associated with striatal degeneration *per se* but with loss of dopamine input to the striatum (Kish et al., 1988; Malapani et al., 1998, 2002). Dopamine is thought to regulate the speed of an internal clock (Artieda et al., 1992; Pastor et al., 1992; Harrington et al., 1998; Buhusi and Meck, 2002; MacDonald and Meck, 2005; Cheng et al., 2007; Jones et al., 2008; Coull et al., 2011) and dopamine levels and responses have been directly implicated in timing control (Meck, 1996; Buhusi and Meck, 2002; Buhusi, 2003; Snowden and Buhusi, 2019). In some studies, intervals were perceived as larger when tonic dopamine levels were reduced (Lake and Meck, 2013; Heilbronner and Meck, 2014; Agostino and Cheng, 2016), and this affects intertemporal choice when subjects choose between different sized rewards at different temporal delays. Phasic dopamine decrease has also been found to reduce the perceived interval duration (Soares et al., 2016) and nigrostriatal dopamine lesions alter timing performance (Meck, 2006).

Dopamine levels are also thought to be important in schizophrenia, where dopamine hyperactivity has been found in the striatum (Meyer-Lindenberg et al., 2002). Schizophrenia also shows timing deficits (Johnson and Peztel, 1971; Densen, 1977; Tysk, 1990; Elvevag et al., 2003; Penney et al., 2005; Drew et al., 2007) associated with BG dysfunction, in particular, caudate hypoactivation occurs during interval timing tasks (Volz et al., 2001). Timing deficits seen in schizophrenia could occur through dysregulation of dopamine D2 signaling in the striatum since patients display increased striatal D2 receptor density (Wong et al., 1986; Laruelle, 1998). Furthermore, transgenic mice overexpressing D2 receptors were impaired in an operant interval timing task (Kellendonk et al., 2006; Drew et al., 2007), while D2 agonists and antagonists alter interval timing performance (Meck, 1986; Drew et al., 2003; Matell et al., 2006; Taylor et al., 2007).

In the future, it will be valuable to model ramping and slow, dynamically critical activity in the two separate but interacting D1 and D2 MSN subsystems including appropriate dopamine modulated cortical-striatal plasticity. Indeed D1 and D2 MSNs are differently involved in cortico-striatal dopamine modulated LTP and LTD (Reynolds et al., 2001; Reynolds and Wickens, 2002). The relative timing of their activations and deactivations (Yagishita et al., 2014; Shindou et al., 2019) is thought to play a crucial role in reinforcement learning and be closely related to interval timing circuits (Meck, 1988, 2006; Coull et al., 2011; Merchant et al., 2013; Petter et al., 2018). MSN to MSN inhibitory network dysfunction between and within D1 and D2 subpopulations could lead to abnormal reinforcement schedules which underly a number of dopamine related disorders of the BG. Since dopamine modulated cortico-striatal plasticity depends on the activity of postsynaptic MSNs and presynaptic cortical neurons, as well as on the local dopamine concentration (Reynolds et al., 2001; Reynolds and Wickens, 2002), the timing of dopamine release with respect to MSN ramping schedules will have a large effect of whether LTP or LTD occurs in each population, and which is dominant. Such a model could be used to understand the effect of pharmacological manipulations of MSN network synaptic parameters, which result in pathological ramping and disrupted network dynamics (Carrillo-Reid et al., 2008; Jáidar et al., 2010), on BG reinforcement learning (Ito and Doya, 2011) and temporal credit assignment, as well as how tonic and phasic dopamine activity affects intertemporal choice and timing tasks. It will also be important to investigate how the temporal discrimination performance of the MSN network model is affected when cortical input drive and dopamine levels are modified in ways that may occur in PD or schizophrenia resulting in abnormal population dynamics (Jáidar et al., 2010).

Accurate time estimation is necessary to support the main function of the BG—the control of action and motor sequences. Many BG diseases affect motor response and motor sequencing leading to a range of movement disorders, including both slow hypokinesia, such as PD, and hyperkinesia, such as HD (Gerfen and Surmeier, 2011). Psychiatric diseases, such as schizophrenia and addiction, also result in movement disorders and have been linked to BG dysfunction (Bernard et al., 2017; Peall et al., 2017; Snowden and Buhusi, 2019). BG lesions can also lead to hypokinetic and bradykinetic movement disorders or the generation of hyperkinetic unintentional movements, including choreatic, dyskinetic, and dystonic movements (DeLong, 1990; Marsden and Obeso, 1994; Groenewegen, 2003). These effects have been thought to occur through imbalances in the direct and indirect BG pathways. For example, chronic tics and stereotyped movements which occur in Tourette's syndrome have been thought to result from a defective suppression mechanism in the basal ganglia (Mink, 2001). Uncordinated motor output could occur if the direct pathway activity is badly mismatched, or not synchronized, with the indirect one so that the thalamocortical system is either abnormally disinhibited or overinhibited (DeLong, 1990; Gerfen and Wilson, 1996). Discordination of slow network generated timescales, including abnormal ramping, between the striatal D1 and D2 subsystems, which differently modulate the direct and indirect pathways, could be a causal factor in such movement disorders (Jáidar et al., 2019).

In running animals, striatal activity correlates with kinematic parameters such as speed and acceleration (Rueda-Orozco and Robbe, 2015), and pharmacological modifications of striatal activity impair the animal's ability to control these kinematic parameters (Miyachi et al., 1997; Mello et al., 2015; Rueda-Orozco and Robbe, 2015; Lopez-Huerta et al., 2021). Ramping firing patterns in the striatum may play a role in the control of kinetic parameters. Deficits in ramping such as may occur by modification of network dynamics which move, the system away from the critical regime would be expected to alter speed and acceleration in testable ways.

In conclusion, we have shown that when a striatal MSN network model is combined with a simple action selection system, ramping activity in good agreement with the experiment can emerge when the dynamics generated by the network are close to critical. The insight obtained from this basic modeling of ramping could pave the way for more detailed modeling of temporal credit assignment including dynamic reward and cortical-striatal plasticity in a more complex reinforcement learning task environment.

## Methods

### Spiking Network Model

The network model is as described in Ponzi and Wickens (2008, 2010, 2013). The network is composed of model MSNs with parameters set so they are in the vicinity of a bifurcation from a stable fixed point to spiking limit cycle dynamical behavior (Ponzi and Wickens, 2008, 2010). These models the dynamics in the UP state when the cells are all receiving excitatory drive to firing threshold levels of depolarization. To describe the cells we use the *I*_*Na, p*_+*I*_*k*_ model described in Izhikevich (2005) which is two-dimensional and given by,


(1) $$\begin{array}{l} C\frac{dV_{i}}{dt}=I_{i}(t)-g_{L}(V_{i}-E_{L})\\ \;-g_{Na}m_{\infty }(V_{i})(V_{i}-E_{Na})-g_{k}n_{i}(V_{i}-E_{k})\\ \;\frac{dn_{i}}{dt}\;=\;(n_{\infty }-n_{i})/\tau_{n} \end{array}$$

having leak current *I*_*L*_, persistent *Na*^+^ current *I*_*Na, p*_ with instantaneous activation kinetic, and a relatively slower persistent *K*^+^ current *I*_*K*_. *V*_*i*_(*t*) is the membrane potential of the *i*^*th*^ cell, *C* the membrane capacitance, *E*_*L, Na, k*_ are the channel reversal potentials, and *g*_*L, Na, k*_ are the maximal conductances. *n*_*i*_(*t*) is *K*^+^ channel activation variable of the *i*^*th*^ cell. The steady state activation curves *m*_∞_ and *n*_∞_ are both described by, $x_{\infty }\left(V\right)=1/\left(1+\exp\left\{\left(V_{\infty }^{x}-V\right)/k_{\infty }^{x}\right\}\right)$ where *x* denotes *m* or *n* and $V_{\infty }^{x}$ and $k_{\infty }^{x}$ are fixed parameters. τ_*n*_ is the fixed timescale of the *K*^+^ activation variable. The term *I*_*i*_(*t*) is the input current to the *i*^*th*^ cell.

All the parameters are set as in Izhikevich (2005) so that the cell is in the vicinity of a *saddle-node on invariant circle* (SNIC) bifurcation, characterizing a Type 1 neuron model. As the current *I*_*i*_(*t*) in Equation 1 increases through the bifurcation point, a limit cycle having zero frequency is formed (Izhikevich, 2005), whose frequency increases slowly with increasing current. This is an appropriate model to use for an MSN in the UP state since its dynamics are in qualitative agreement with several aspects of MSN firing (Ponzi and Wickens, 2013).

The input current $I_{i}\left(t\right)=I_{i}^{M}\left(t\right)+I_{i}^{C}\left(t\right)$ in Equation 1 is composed of two parts. Component $I_{i}^{M}\left(t\right)$ is the inhibitory feedback term that comes from the recurrent collaterals of the MSN inhibitory network and component $I_{i}^{C}\left(t\right)$ represents the current from excitatory feedforward sources, the cortex, and the thalamus (refer to below). In the real striatum, another component of the input current would come from other cells such as fast spiking interneurons (FSIs) which are thought to be mainly feedforward. Studies (Mallet et al., 2005; Gittis et al., 2010) have shown that fast-spiking interneurons are strongly connected to spiny projection neurons and mediate the bulk of feedforward inhibition. Activation of cortical afferents excites fast-spiking interneurons approximately 2–6 ms earlier than spiny projection neurons (Mallet et al., 2005) and a lesion of fast-spiking interneurons impair the acquisition of sequence learning strategies (Owen et al., 2018). This FSI component of the input current is not included in the present model, although its feedforward component can be considered simply as a constant offset term to be included in the feedforward driving $I_{i}^{C}\left(t\right)$. Similarly, we do not consider secondary feedback effects MSNs may have on each other *via* other more complex secondary pathways such as *via* other cell types in the striatum or *via* cells in other nuclei such as the Globus Pallidus, dopaminergic systems, or the cortex.

The MSN network synapses are described by Rall-type synapses (Rall, 1967) and the input current is given by, $I_{i}^{M}\left(t\right)=-\left(V_{i}\left(t\right)-V_{M}\right)\overset{N}{\underset{j}{\sum }}k_{ij}^{M}g_{ij}\left(t\right)$. The input current to a postsynaptic neuron *i* is summed over all inhibitory presynaptic neurons *j* where *N* = 500 is the number of cells in the network simulation and *V*_*M*_ = −65*mV* is the synaptic reversal potential. *g*_*ij*_(*t*) is the quantity of neurotransmitter bound to postsynaptic cell *i* emitted from presynaptic cell *j*. It is given through, $\tau_{g}\frac{dg_{ij}}{dt}=\Theta \left(V_{j}\left(t\right)-V_{th}\right)-g_{ij}$. Here, *V*_*th*_ = −40*mV* is a threshold. τ_*g*_ is a timescale that has been adjusted so that the IPSP decay time scale is near that observed in experimental studies (Ponzi and Wickens, 2010, 2013). In simulations, here, we use the value τ_*g*_ = 50 so that postsynaptically bound neurotransmitter exponentially decays to half its value in time τ_*g*_*ln*(2)≈34 ms. Θ(*x*) is the Heaviside function. Since the initial value of the neurotransmitter *g*_*ij*_(0) decays exponentially with timescale τ_*g*_, then *g*_*kj*_(*t*) = *g*_1*j*_(*t*) for all *k* at times *t*>>τ_*g*_ and we only need to keep track of a single *g*_*j*_(*t*) for each cell *j*. The inhibitory current into a postsynaptic cell *i* is then,


(2) $$\begin{array}{r} I_{i}^{M}\left(t\right)=-\left(V_{i}\left(t\right)-V_{M}\right)\underset{j}{\sum }k_{ij}^{M}g_{j}\left(t\right), \end{array}$$

and *g*_*j*_ is simply an exponentially weighted moving average of cell *j* firing, given by,


(3) $$\begin{array}{r} \tau_{g}\frac{dg_{j}}{dt}=\Theta \left(V_{j}\left(t\right)-V_{th}\right)-g_{j}. \end{array}$$

The representation of the MSN network is determined by the synaptic strengths of $k_{ij}^{M}$ in Equation 2. They are given by,


(4) $$\begin{array}{r} k_{ij}^{M}=\left(k^{M}/\rho \right)\epsilon_{ij}Z_{ij}. \end{array}$$

Here, ϵ_*ij*_ in Equation 4 is a uniform quenched random variable drawn from the interval [0.8, 1.2] independent in *i* and *j* so that the expectation 〈_ϵ_*ij*_〉*ij*_ = 1, which produces a more realistic random distribution of connection strengths. Thus, even reciprocally connected cells have asymmetric inhibition, (ϵ_*ij*_−ϵ_*ji*_≠0), as is the case in reality.

*Z*_*ij*_ is a parameter that takes the value *Z*_*ij*_ = 1 if cells *i* and *j* are connected and zero otherwise. ρ = 〈_*Z*_*ij*_〉*ij*_ is the network connection probability. Since MSN network structure within a local striosome does not indicate anything other than a random process of connection growth we connect pairs of cells randomly with probability ρ which generates networks with binomial degree distributions. There are no self-connections, *Z*_*ii*_ = 0. ρ is the main parameter varied in the simulations to generate different dynamics ranging from stable to chaotic, as described in Ponzi and Wickens (2013). *k*^*M*^ is a fixed peak conductance parameter. The level of inhibition onto an MSN is held approximately constant as ρ is varied by the rescaling of *k*^*M*^ by ρ in Equation 4. ρ for the real striatum is expected to be around 0.16 within a 500 cell local network as described in Ponzi and Wickens (2013). The value of *k*^*M*^ is set so that IPSPs are around 200μ*V*, very similar to real striatal IPSPs, at connectivities of around ρ = 0.16 when the postsynaptic cell is just above the firing threshold. Here, we investigate network simulations with connection probabilities ρ between 0.04 and 0.4 in increments of 0.0025.

We model the excitatory driving $I_{i}^{C}\left(t\right)$ as a stochastic process, as described in Ponzi and Wickens (2013), $I_{i}^{C}\left(t\right)=\left(V_{C}-V_{i}\left(t\right)\right)X_{i}\left(t\right)$. *V*_*C*_ is the excitatory reversal potential, set here to 0.0 mV. In general, the excitatory component will also be given by Rall type synapses (Rall, 1967; Destexhe et al., 1998). Therefore, we calculate *X*_*i*_(*t*) using,


(5) $$\begin{array}{c} \tau_{a}dX_{i}=(\sum_{l}^{N_{C}}b_{il}r_{il}^{S}-X_{i})dt+\epsilon_{i}(t){\left[dt\sum_{l}^{N_{C}}{\left(b_{il}\right)}^{2}r_{il}^{S}\right]}^{1/2}. \end{array}$$

ϵ_*i*_(*t*) is a random variable independent in both cell *i* and time *t*. The *b*_*il*_ is the maximal conductance parameter from the *l*^*th*^ excitatory cortical or thalamic input to the *i*^*th*^ MSN cell. They are fixed in our simulations reported here. We assume the inputs from each of the *N*_*C*_ input cells follow independent Poisson process with rates $r_{il}^{S}$ which are fixed for a given stimulus *S*. MSN cells are each contacted by around 10,000 cortical and thalamic cells and we, therefore, set *N*_*C*_ = 10, 000 in Equation 5. These excitatory inputs *l* are considered to be non-overlapping between the MSN cells *i*. Our assumption of zero common input is not, however, supposed to be a statement of biological fact. We wish to investigate how correlated activity arises from local interactions among MSNs, rather than *via* common input.

Here, we investigate how the MSN network model responds to a simple kind of temporally varying cortical input. This is just a sequence of different stimuli. To model this we simply change all the cortical input rates *r*_*il*_ suddenly, hold them fixed for a period of time, then change them again suddenly. Each given set of rates $r_{il}^{S}$, held fixed for a period of time, is denoted a stimulus, *S*. In the simulations reported here we generate two stimuli $r_{il}^{A}$ and $r_{il}^{B}$ which are then applied alternately with variable length intervals. One stimulus represents the cue and time out stimulus the other is a background stimulus applied in between applications of the cue/time out stimulus during the trial period and inter trial interval. The second stimulus represents background noise. The two stimuli are identical statistically, we do not need to make the cue stimulus stronger or more salient.

For each stimulus *S*, the 10, 000×*N* input rates $r_{il}^{S}$, where *N* = 500 is the number of MSN cells, are drawn independently from a fixed distribution, a normalized Pareto distribution, $f_{\gamma ,\alpha }\left(x\right)=\gamma \alpha /{\left(1+\gamma x\right)}^{1+\alpha }$, with tail parameter α and expectation 1/(γ(α−1)). The normalized Pareto distribution with power-law tail parameter α < 2 is chosen so that even though there are many, 10,000, inputs to each cell the mean input strength can still show large fluctuations across cells. If instead the *r*_*il*_ is chosen from a narrow distribution, e.g., the Gaussian, when many inputs are averaged, all the cells will have approximately the same input strength, and stimulus specificity will not be generated. We have chosen to use the Pareto distribution simply as a simple way to produce a large variation in excitation strength across MSN cells.

Here, we do not vary the parameters of the Pareto distribution α and γ and set α = 1.75 in all simulations. γ is set so that the input rates *r*_*il*_ have the expectation of 0.02 spikes per ms (due to the Pareto distribution the fluctuations across cells around this mean value will be large). We choose all the channel parameters *b*_*il*_ independently from a uniform distribution on [0, 2*b*]. The parameter *b* is set so the expectation of *b*_*il*_ is *b* = 0.0006. These parameters result in a mean input current of 0.32 nA with a SD of temporal fluctuations in input current of 0.0053 nA. If a cell *j* has a mean input current below the firing threshold 0.2 nA its *r*_*jl*_ and *b*_*jl*_ are redrawn until otherwise. Thus, all cells are driven above the firing threshold by cortical excitation.

All simulations were carried out with the stochastic weak second order Runge-Kutta integrator described in Burrage and Platen (1999) with an integration time step of 0.1 ms.

### Matching rate network and Lyapunov exponent calculation

Maximal Lyapunov exponents are calculated from a matching rate network model, as described in Ponzi and Wickens (2013).

The matching rate model is obtained from the equation for the postsynaptically bound neurotransmitters *g*_*j*_, Equation 3, by replacing the Heaviside function Θ(*V*(*t*)−*V*_*thr*_) with *Tδ*(*t*_*i*_). δ() is the Dirac delta function and *t*_*i*_ are spike times. *T* is the time period the membrane potential *V*(*t*) exceeds the threshold *V*_*thr*_ = −40*mV* during a spike, which turns out to be *T*≈1 ms for this cell model. This approximation is valid, here, since *T* < < τ_*g*_. Then δ(*t*_*i*_) is replaced by cell *j* spiking probability per ms, or firing rate, at time *t*, *F*_*j*_(*t*) to obtain,


(6) $$\begin{array}{r} \tau_{g}\frac{dg_{j}}{dt}=TF_{j}\left(t\right)-g_{j}. \end{array}$$

*F*_*j*_(*t*) is determined by the cell's input current to give,


(7) $$\begin{array}{r} \tau_{g}\frac{dG}{dt}=-G+Ts{\sqrt{\left(V_{C^{\prime }}X-V_{M^{\prime }}KG-I_{bif}\right)}}_{+} \end{array}$$

where *G* = {*g*_1_, *g*_2_, ...*g*_*N*_} are the postsynaptically bound neurotransmitters for the *N* = 500 cells. $K=\left\{k_{ij}^{M}\right\}$ is the fixed connection matrix, determined exactly as in the simulations for the full model. *X* = {*X*_*i*_}, $X_{i}=\overset{N_{C}}{\underset{l}{\sum }}b_{il}r_{il}$ are the expected values of the excitatory inputs determined exactly as in the full model, neglecting the noise term. $V_{C^{\prime },M^{\prime }}$ are fixed scalar parameters accounting for the conductance based synapses. These are set as the difference between the resting potentials and reversal potentials, $V_{C^{\prime }}=60.0$ mV for excitatory synapses and $V_{M^{\prime }}=5.0$ mV for inhibitory synapses. τ_*g*_ = 50 ms as in the full simulations. The function $s{\sqrt{\left(x\right)}}_{+}$, ($s\sqrt{x}$ for *x*>0 and zero otherwise), is the dependence of firing rate on input current for Type 1 cells and the parameter *s* is estimated from the current vs. firing rate plot for these cells to be *s* = 0.09. *I*_*bif*_ = 0.2 nA is the current at the firing threshold.

The maximal Lyapunov exponent is calculated in the standard way, as described in Ponzi and Wickens (2012), from the rate network dynamical evolution. First, a perturbed 500 dimensional orbit, $\vec{G_{p}\left(0\right)}$, near the system orbit, $\vec{G\left(0\right)}$ is selected by random perturbation *d*_0_ = *G*_*p*_(0)−*G*(0) of size $D_{0}=\surd d_{0}^{2}=10^{-12}$. Both orbits are iterated once for time Δ*t* and the new separation vector is calculated, *d*_1_ = *G*_*p*_(Δ*t*))−*G*(Δ*t*)), with length $D_{1}=\surd d_{1}^{2}$. The maximal Lyapunov exponent is calculated as λ_1_ = *ln*(*D*_1_/*D*_0_)/Δ*t*. The perturbed orbit is rescaled in the direction of maximal separation, *G*_*p*_(0)←*G*(Δ*t*))+*d*_1_(*D*_0_/*D*_1_). This process is repeated for many iterations and many values λ_*j*_ = *ln*(*D*_*j*_/*D*_0_)/Δ*t* of the Lyapunov exponent are calculated. The average is calculated after discarding a long transient sufficient for the perturbation to be in the maximally separating direction.

Numerical integrations of the deterministic rate network are performed using a fourth-order Runge-Kutta.

### Task environment

The task environment follows Gouvea et al. (2015). Task stimuli are modeled as different excitatory inputs (from cortex or thalamus) to the MSN inhibitory network. There are two inputs, one is a cue stimulus and one is the background input applied whenever the cue is not. This background input may be considered background lab sensory input irrelevant to the task, or simply excitation from the cortex arising internally. The MSN network is purely inhibitory so some form of excitation is needed if there is to be any activity at all.

Both excitatory stimuli are randomly drawn from the same distribution as described above. They are statistically identical. The cue stimulus is not stronger or more salient than the background stimulus. As in Gouvea et al. (2015) the cue stimulus is a 150 ms square pulse applied twice, once at the start of a trial interval and once at the end of the trial interval. Between the applications of the cue stimulus is the trial interval. For each trial, the trial interval is drawn with uniform probability from 600, 1,050, 1,260, 1,380, 1,620, 1,740, 1,950, and 2,400 ms. Between trials, after the end of the second cue stimulus, there is a fixed time-out of 600 ms followed by a further exponentially distributed interval of mean 200 ms.

In each network simulation, successive trials are drawn randomly from the set of eight possible trial lengths. Each trial, therefore, has a 50% chance of being long or short. Simulations have a fixed length of 3,37,680. Trials have variable lengths depending on which interval was chosen and on the random length of the inter trial interval. There are roughly 130 trials in a network simulation, so there are roughly 65 long and 65 short. As in Gouvea et al. (2015), we did not attempt to fix the quantity of long and short trials to balance exactly.

### Action selection and Correct Response Probability (CRP)

Action selection is implemented in the same way as Gouvea et al. (2015).

First rate time series for individual cells are calculated from the spiking network as follows. A 10,000 ms transient is discarded. All cells which do not spike once during the simulation are discarded. Spikes for each cell are assigned to non-overlapping 10 ms bins. Only the 50 cells with the highest average firing rate are used in action selection (this number can vary but the number of cells used should be smaller than the number of trials for the Linear Discriminant Analysis to be applied).

Trial average rates are calculated for each of these cells. As in Gouvea et al. (2015) this is the average firing rate in the final 500 ms of each trial of M trials. This results in a 50 dimensional vector for each trial in the simulation and the trial classification as short (< 1, 500 ms) or long (> 1, 500 ms) for each trial. Next, the Fischer Linear Discriminant (FLD) analysis is applied M times as in Gouvea et al. (2015) to this data set. Each time one trial is left out. The maximally discriminative vector (MDV) is calculated from the remaining M-1 trials. This is the vector $\vec{w}$ that maximizes,


(8) $$\begin{array}{r} L\left(\vec{w}\right)=\frac{{\left(\vec{w}\cdot \left(\vec{\mu_{L}}-\vec{\mu_{S}}\right)\right)}^{2}}{{\vec{w}}^{T}\left(\Sigma_{L}+\Sigma_{S}\right)\vec{w}} \end{array}$$

where $\vec{\mu_{L}}$ and $\vec{\mu_{S}}$ are the mean vectors for the long and short trials, respectively, and Σ_*L*_ and Σ_*S*_ are the corresponding covariance matrices. The discrimination criteria *c* is given by the projection of the mean of the long and short trials onto $\vec{w}$,


(9) $$\begin{array}{r} c=\vec{w}\cdot \left(\vec{\mu_{L}}+\vec{\mu_{S}}\right)/2. \end{array}$$

To determine the action generated for the remaining trial, its activity $\vec{R}$ is projected onto $\vec{w}$ and compared with *c*, $\vec{R}\cdot \vec{w}>c$ for a long trial. This leave-one-out procedure is repeated to generate a selected action for each trial.

The MDV $\vec{w}$ was computed using the ALGLIB library in C++. https://www.alglib.net.

Correct response probabilities CRP are calculated for intervals separately, the 600 and 2,400 ms intervals are combined and denoted CRP_4_, the 1,050 and 1,950 ms intervals are combined and denoted CRP_3_, the 1,260 and 1,740 ms intervals are combined and denoted CRP_2_, and the 1,380 and 1,620 ms intervals are combined and denoted CRP_1_, i.e., for each trial *j* in a given simulation we obtain an action selection *A*_*j*_ = 1, 0 if the action is long or short, respectively. Each trial also has an interval length denoted *I*_*j*_, CRP_1_ is given by,


(10) $$\begin{array}{c} CRP_{1}=\frac{\sum_{j}\delta_{I_{j},1380}(1-A_{j})+\sum_{j}\delta_{I_{j},1620}A_{j}}{\sum_{j}\delta_{I_{j},1380}+\sum_{j}\delta_{I_{j},1620}} \end{array}$$

where δ_*x, y*_ = 1 if *x* = *y* and zero otherwise, and similarly for the other CRPs.

### Preference indices

Cell preferences are determined for all cells that fire at least one spike (not just the 50 used in action selection) using receiver operating characteristic (ROC) analysis as in Gouvea et al. (2015). First, the action selected for each trial are determined, as described above. The distributions of firing rates in the last 500 ms of trials is determined across long selected trials and across short selected trials for each cell. For each cell, a ROC value is calculated using these distributions in the standard way. That is, for a given cell the mean firing rate in the final 500 ms of each trial is calculated for long and short selected trials separately. For each trial type, the firing rates are binned in 1 Hz bins and the probability of each bin is obtained by dividing by the number of trials of that type, *M*_short_ and *M*_lomg_. Next, a threshold firing rate, *T*, is defined. The total cumulative probability below the threshold, *T*, for the short trials (true positives) and the long trials (false positives) is obtained. This is repeated for all thresholds from zero incremented in 1 Hz steps. The pair of probabilities (prob_short_, prob_long_) for each threshold value, *T*, forms a curve starting at (0,0) and increasing to (1,1). The area under this curve, *A*, provides a measure of how separate the distributions are, with unity being strongly long preferring and zero being strongly short preferring. Here to calculate preference indices we generate a z-score. We draw with replacement *M*_short_ observations and *M*_long_ observations from the firing rates for the cell across all trials. Using these distributions a surrogate ROC area, *A*_surr_ is obtained. This is repeated 50 times. Finally, the preference index z-score is obtained as *A*_*Z*_ = (*A*−〈*A*_surr_〉)/σ(*A*_surr_) where 〈*A*_surr_〉 denotes the mean over the surrogate *A*_surr_ values and σ(*A*_surr_) is their SD.

Averages over significantly long preferring cells and significantly short preferring cells are determined as averages over cells with *A*_*Z*_ greater than 1 and less than –1, respectively.

## Data Availability Statement

The raw data supporting the conclusions of this article will be made available by the authors, without undue reservation.

## Author contributions

AP and JW contributed to the conception, design of the study, discussed the results, and edited the final version of the manuscript. AP wrote the code, performed simulations, and wrote the first draft of the manuscript. Both authors read and approved the submitted version.

## Funding

European Union's Horizon 2020 Framework Programme for Research and Innovation under the Specific Grant Agreement numbers 945539 (Human Brain Project SGA3). Subsidy funding from the Okinawa Institute for Science and Technology Graduate University.

## Conflict of interest

The authors declare that the research was conducted in the absence of any commercial or financial relationships that could be construed as a potential conflict of interest.

## Publisher's note

All claims expressed in this article are solely those of the authors and do not necessarily represent those of their affiliated organizations, or those of the publisher, the editors and the reviewers. Any product that may be evaluated in this article, or claim that may be made by its manufacturer, is not guaranteed or endorsed by the publisher.
